# Effect of sample management on quantitative HIV-1 viral load measurement at Saint Paul’s Hospital Millennium Medical College, Addis Ababa, Ethiopia

**DOI:** 10.1371/journal.pone.0269943

**Published:** 2022-06-14

**Authors:** Gadissa Gutema, Habtyes Hailu, Belete W/semeyat, Amelework Yilma, Saro Abdela, Eleni Kidane, Sisay Adane, Mengistu Yimer, Aster Tsegaye

**Affiliations:** 1 HIV/AIDS Disease Research Team, TB and HIV/AIDS Disease Research Directorate, Ethiopian Public Health Institute, Addis Ababa, Ethiopia; 2 Department of Medical Laboratory Sciences, College of Health Sciences, Addis Ababa University, Addis Ababa, Ethiopia; 3 TB Disease Research Team, TB and HIV/AIDS Disease Research Directorate, Ethiopian Public Health Institute, Addis Ababa, Ethiopia; "INSERM", FRANCE

## Abstract

**Purpose:**

This study was meant to determine the effect of time to plasma separation, storage duration, freeze-thawing cycle and dilution proportion on the HIV-1 viral load level.

**Methods:**

Experimental study design was employed by collecting 10mL whole blood samples into two EDTA tubes from 88 eligible HIV infected patients at St Paul’s Hospital Millennium Medical College. The viral load test was done using Abbott m2000sp/rt analyzer. Data was entered into Microsoft excel and analyzed by SPSS version 20. Repeated measure analysis of variance was used to compare HIV RNA viral load mean difference between different time to plasma separation, storage, freeze-thawing cycles and dilution levels. Post-hoc analysis was employed to locate the place of significant differences. P value less than 0.05 was used to declare statistical significance while viral RNA level of 0.5 log copies/ml was used to determine clinical significance.

**Results:**

There was significant HIV-1 RNA viral load log mean difference between plasma separation time at 6 hours (hrs) and 24hrs (*p<0*.*001*). There was also significant HIV-1 RNA viral load log mean difference between plasma tested within 6hrs and those stored at 2–8°C for 15 days (*p = 0*.*006)*, and between plasma stored at 2–8°C for 6 days versus 15 days (p*<0*.*001*). There was significant log mean difference between plasma that was exposed to fourth cycle of freeze-thawing after storage at -20°C when compared with plasma tested within 6hrs (*p = 0*.*013*).

**Conclusion:**

Plasma separated at 24hrs, stored at 2–8°C for 15 days or freeze-thawed for four cycles had significant effect on HIV viral load level. However, the differences were not clinically significant at a cut-off viral load level of 0.5 log copies/ml. Avoiding delays to plasma separation beyond 24 hrs, storing at 2–8°C for 15 days and freeze-thawing for no more than 4 cycles is recommended to improve the result quality.

## Introduction

Human immunodeficiency virus (HIV)/Acquired immunodeficiency syndrome (AIDS) disease progression and antiretroviral therapy (ART) success are monitored by Cluster of Differentiation 4 (CD4) T-cell quantification, clinical criteria and viral load quantification [1]. Although it is not widely accessible in resource limited countries, viral load quantification is the gold standard in the detection of HIV-1 disease progression and treatment failure [2, 3].

The reproducibility and accuracy of HIV-1 Ribonucleic Acid (RNA) load quantification are still major concerns, because HIV-1 RNA stability in whole blood or plasma is affected by several factors [4, 5]. Quality specimen handling prior to HIV-1 RNA concentration testing is important as poor specimen handling could affect the detection and quantification of the virus [5, 6]. There is contradicting evidence regarding whether values of HIV-1 RNA obtained under different conditions vary significantly [7]. For example, previous studies indicated that, plasma sample can stay at room temperature without viral degradation for up to 24 hours [2, 8, 9], 5 days at 2–8°C and for longer periods at -80°C [2, 8].

On the other hand, a decline in HIV-1 RNA concentration has been observed when samples are analyzed after whole blood is stored for 24hrs, although the differences are not statistically significant [10, 11]. In contrast, certain studies have shown that HIV-1RNA is stable for up to 72hrs at room temperature in whole blood sample [12–14].

There is evidence that shows HIV-1 RNA in plasma specimens stored at 4°C for 1 week does not affect HIV-1 RNA measurement when compared with HIV-1 RNA concentrations determined from fresh plasma [15–17]. It was also found that HIV-1 RNA copy number remained within 0.5 log copies/ml (clinically significant viral load level) when plasma is stored at 4°C for one week, with a viral load difference of less than 0.5log or ±2SD difference considered as normal assay variation of plasma RNA level. Viral load changes of greater than 0.5log or ±2SD indicates significant clinical difference [13]. It has been demonstrated that plasma samples held at room temperature for up to three days [12] as well as up to 14 days [15] did not show clinically significant difference. In contrast, HIV-1 RNA levels decreased significantly when plasma was stored at 37°C for one week [15].

Frequency of freeze-thaw cycle is also among the factors that affect the stability of HIV-1 RNA [12]. Direct and close association between freeze-thaw cycle and HIV-1 viral load has been demonstrated [12]. However, there is evidence indicating the absence of significant effect of plasma freeze-thawing on HIV-1 viral load [18, 19]. HIV-1 RNA was also stable in plasma stored at -20°C and -70°C despite three freeze-thaw cycles [12, 20].

In practice, plasma could be diluted by diluents when the volume is insufficient or when the concentration of HIV-1 RNA is above the reading limit of a given instrument. However, plasma dilution by phosphate buffered saline (PBS) has been shown to have an effect on the HIV-1 RNA concentration [21].

Laboratory infrastructure, sample transportation and storage problems are the main challenges that affect HIV-1 RNA viral load quantification in developing countries [22, 23]. Ethiopia is among high HIV prevalent countries with a recent national prevalence of 0.9% [24] and according to the latest spectrum modeling, an estimated 610,335 people were living with HIV in 2018 [25]. Ethiopia has introduced routine HIV-1 viral load testing, the preferred monitoring tool for diagnosing and confirming ART failure [26] based on WHO recommendation [27]. The country also adopted the UNAIDS 90–90–90 by 2020 target [28]. Viral load testing is a key component of achieving and monitoring progress towards the “third 90” target. The country follows a sample referral linkage system strategy to scale up the routine viral load monitoring service. However, poor specimen handling and poor referral linkage system could result in RNA degradation which may lead to incorrect reporting of unsuppressed HIV-1 viral load results as suppressed [27]. This may lead to drug regimen shift delays, resulting in the dissemination of resistant strains. This may also mislead policy makers and other stakeholders who hence assume good viral suppression in the population [28]. Although there are different contradicting reports on the effect of plasma separation time, storage, freeze-thaw cycle and dilution on HIV-1 viral load, there is limited evidence to clarify their effects. Ethiopia is one of countries in which such kind of evidence is lacking to support the program.

## Methods

An experimental study design was employed to determine the effect of time to plasma separation, storage temperature, duration of storage, freeze-thaw cycles and dilution proportion on HIV 1 RNA concentration from April to July, 2019 GC. The study was conducted on people living with HIV who have been receiving antiretroviral therapy (ART) for at least six months at St Paul’s Hospital Millennium Medical College in Addis Ababa, Ethiopia.

### Sample size determination and sampling method

Sample size was determined by two means with equal sample size comparison formula by considering 4.1 mean log copies/ml of HIV-1 viral load of plasma stored at room temperature for 24hrs [12] and 3.9 mean log copies/ml of HIV-1 viral load of plasma stored for 48hrs at room temperature [12]. In addition, 80% estimation power, 95% confidence level and 0.2 log difference [12] were considered in sample size calculation. Thus, a total of 88 participants were enrolled to this study without employing any special sampling techniques. Thus, all eligible individuals living with HIV and attending an ART program at the hospital during the study period were included consecutively.

### Specimen and data collection

**Fig 1:**
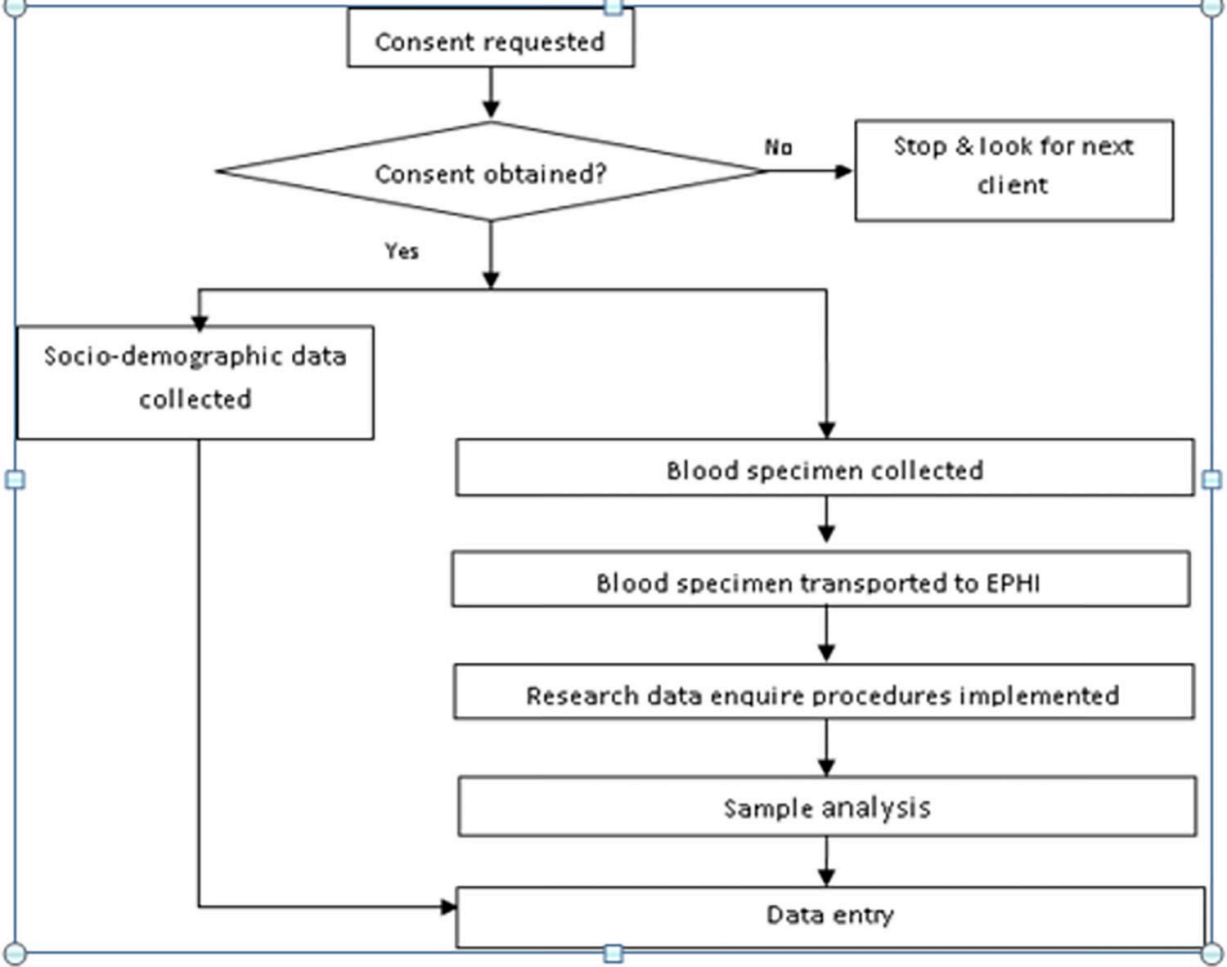
Specimen and data collection flow chart.

Fig 1 depicts the overall procedure of specimen collection. Whole blood samples were collected by the hospital phlebotomists and transported to the molecular laboratory of the Ethiopian Public Health Institute (EPHI) using triple packaging and in temperature monitored condition within five to ten minutes after collection. Standard operating procedure (SOP) was used to follow the sample quality, storage temperature, time of sample collection, sample dilution and freeze-thaw cycles. SOP and Job aids were prepared for each procedure of testing and employed during laboratory operation processes.

### Experiment and laboratory analysis

**Fig 2:**
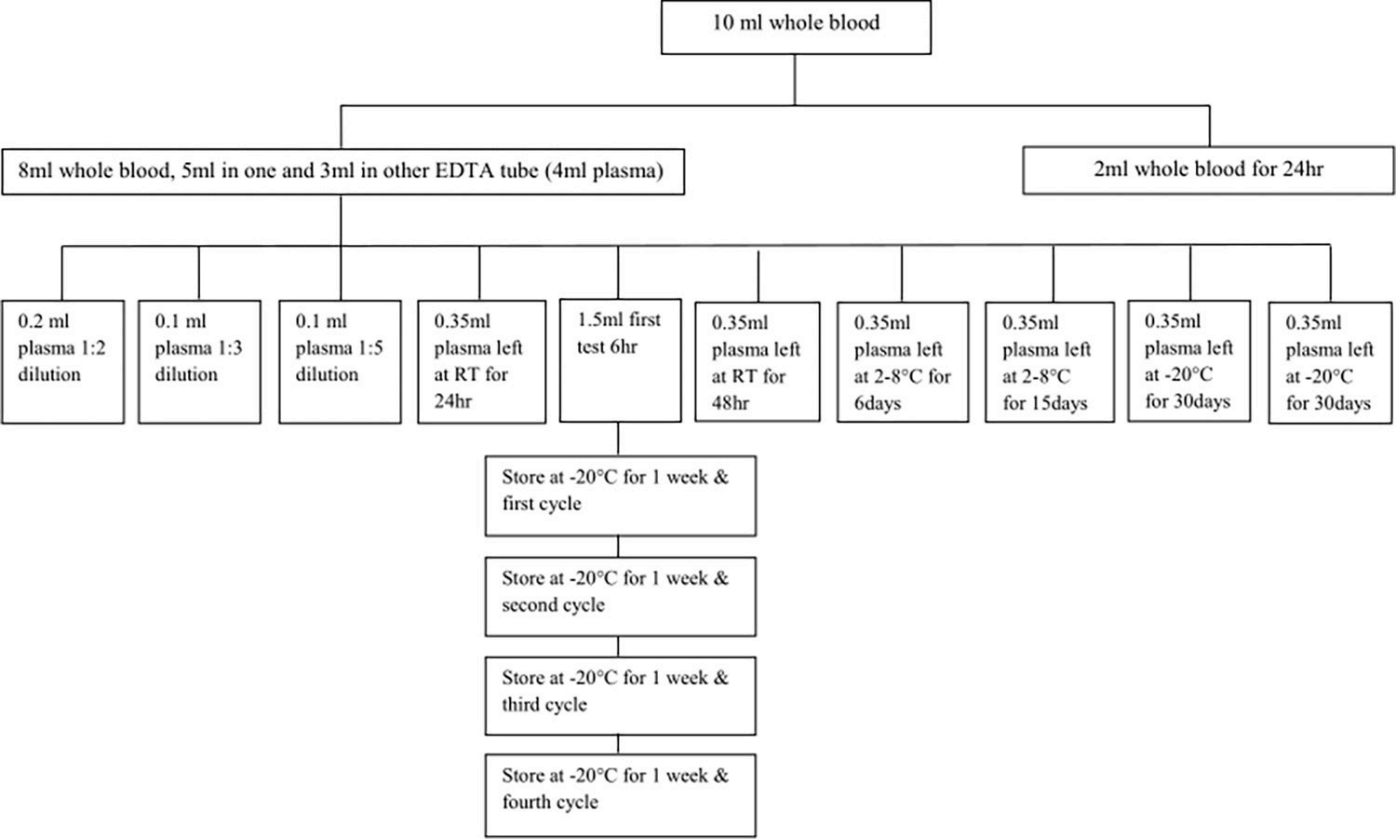
Sample allocation flow chart for each experiment.

Fig 2 shows sample allocation for each experiment. Approximately 10mL of whole blood was collected into two 5ml EDTA tubes. Of these, 2ml was transferred into another EDTA tube and was left at room temperature for 24hrs. The remaining 8ml blood was centrifuged at 5000rpm for 5minutes and 0.35ml plasma was aliquoted into six 1.8mL Thermo Scientific Nunc^TM^ tubes. The six plasma aliquots were stored in three different temperature conditions: two were stored at room temperature, two at 2–8°C and two at -20°C. The remaining one aliquot with 0.4ml volume was diluted in the following concentration ratios: 1:2, 1:3 and 1:5 based on standard practice at EPHI. Results obtained from the diluted samples were multiplied by the dilution correction factors before data analysis was conducted. All diluted plasmas were tested within six hours of collection. The remaining aliquot of 1.5ml was used for a four cycle freeze-thaw experiment. The Real Time HIV-1 Viral Load assay, tested on the m2000 sp/rt platform (both Abbott Molecular, Des Plains, IL, USA), was used to measure the HIV-1 viral load according to the manufacturer’s instructions for use. [29]. Regarding laboratory quality assurance, the Ethiopian National HIV reference laboratory of EPHI participates in proficiency testing (PT) by One World Accuracy (OWA) three times a year and twice per year in the Center for Disease Control (CDC, Atlanta, GA, USA) PT program. The results of PT were acceptable for the quarter in which our experiment was done.

### Data analysis

The data was entered into Microsoft office Excel window 10 and analyzed using SPSS version 20. Data was checked for normality, linearity, outliers and homogeneity of variance-covariance matrices prior to analysis. Descriptive statistics were employed to explain the distribution of socio-demographic characteristics and HIV viral load copies. Repeated Measure Analysis of Variance (RANOVA) was done to determine the mean difference within each experimental scenario. Post-hoc analysis was employed to locate the place of significance within each category if significant difference was observed by RANOVA. The level of significance was set at 5%. Mean differences >0.5 log copies/ml or >±2SD were used as clinical significance threshold [13, 21]. Mean viral load log and RNA copies were used for comparison in all analyses.

### Ethical clearance

Ethical clearances were obtained from the Departmental Research and Ethics Review Committee (DRERC) of the Department of Medical Laboratory Sciences, College of Health Sciences, Addis Ababa University (DRERC/411/19/MLS), and Institution Review board of Ethiopian Public Health Institute (EPHI-IRB-100-2018). All participants provided written informed consent after they understood the objectives and procedures of this study.

## Results

### Characteristics of study participants

A total of 88 PLWHIV were included in this study. Sixty eight percent of the participants were female. The mean (± SD) age of the participants was 37.3 (±8.44) years with the age range of 18 to 53 years. The mean viral load was 4.24 log copies/ ml for the plasma tested at 6hrs after specimen collection, 4.23 log copies/ ml for the plasma stored at 2–8°C for six days and 4.21 log copies/ ml for the plasma stored at -20°C for 30 days [Table 1].

**Table 1:** Mean and median log copies/ml viral load distribution of participants with different sample handling conditions at Saint Paul’s Hospital Millennium Medical College, 2019.

Variable	Mean (±SD)	Median (IQR)
HIV viral load at 6hr	4.24(0.82)	4.25(3.53–4.94)
HIV viral load 1 week freeze thaw first cycle	4.24(0.83)	4.25(3.56–4.93)
HIV viral load 1 week refreeze thaw second cycle	4.2(0.89)	4.13(3.5–4.94)
HIV viral load 1 week refreeze thaw for third cycle	4.22(0.86)	4.20(3.54–4.92)
HIV viral load 1 week refreeze thaw for fourth cycle	4.18(0.80)	4.2(3.51–4.84)
HIV viral load results for plasma stored at RT for 24hrs	4.22(0.81)	4.19(3.6–4.9)
HIV viral load results for plasma stored at RT for 48hrs	4.20(0.81)	4.15(3.6–4.91)
HIV viral load result of WB separated after 24hrs	4.1(0.83)	4.06(3.42–4.8)
HIV viral load results for plasma stored at 2–8°C for 6days	4.23(0.84)	4.25(3.6–4.91)
HIV viral load results for plasma stored at 2–8°C for 15 days	4.18(0.85)	4.1(3.59–4.9)
HIV viral load results for plasma stored at -20°C for 30 days	4.21(0.83)	4.03(3.67–4.9)
HIV viral load results for plasma stored at 2–8°C for 60 days	4.20(0.86)	4.16(3.59–4.84)
HIV viral load result of 1 to 2 dilution(RNA copies)	3.98(0.79)	3.94(3.42–4.56)
HIV viral load result of 1 to 3 dilution (RNA copies)	3.83(0.8)	3.85(3.16–4.41)
HIV viral load result of 1 to 5 dilution (RNA copies)	3.61(0.79)	3.64(3.02–4.2)

RT-Room temperature, WB-Whole blood, SD-Standard deviation, IQR-Inter quartile range, HIV-Human Immunodeficiency virus and RNA-Ribonucleic Acid

### Viral load level at different plasma separation time

There was a significant difference between mean viral load tested within 6hrs of specimen collection and from plasma separated after 24hrs (*p < 0*.*001*). Lower mean (± SD) viral load was observed when the sample was stored as whole blood for 24hrs as compared to viral loads tested within 6hrs (4.1±0.83 versus 4.24±0.82) [Table 2]. The mean difference was 0.14 log copies/ml, which was not clinically significant at the recommended cut-off point of 0.5 log copies/ml.

**Table 2:** Repeated measurement analysis of variance in log/ml for each plasma separation time, storage at room temperature, 2–8°C and -20°C.

Variables	Measurement Indices			
	Mean(±SD) log/ml copies	Mean Difference	F- Statistics	*P- value*
HIV VL within 6hrs—HIV VL Separated after 24hrs (log copies/ml)	4.24(0.82) - 4.1(0 .83)	0.145	33.11	**<0.001**
Within group comparison HIV VL for plasma stored for different time			1.106	0.336
HIV VL within 6hrs—HIV VL 24hrs RT	4.24(0.82) -4.22(0.81)	0.025	1.106	0.999
HIV VL within 6hrs—HIV VL 48hrs RT	4.24(0.82) -4.20(0.81)	0.038	1.106	0.430
HIV VL 24hrs RT—HIV VL 48hrs RT	4.22(0.81)-4.20(0.81)	0.013	1.106	0.999
Within group comparison of (log copies/ml)			10.164	**<0.001**
HIV VL within 6hrs- HIV VL 2–8°C for 6 days	4.24(0.82)- 4.23(0.84)	0.007	10.164	0.999
HIV VL within 6hrs- HIV VL 2–8°C for 15 days	4.24(0.82)-4.18(0.85)	0.060	10.164	**0.006**
HIV VL 2–8°C for 6 days- HIV VL 2–8°C for 15 days	4.23(0.84) -4.18(0.85)	0.052	10.164	**<0.001**
Within group comparison of (log copies/ml)			0.554	0.576
HIV VL within 6hrs- HIV VL -20°C for 30 days	4.24(0.82)-4.21(0.83)	0.029	0.554	0.899
HIV VL within 6hrs- HIV VL -20°C for 60 days	4.24(0.82)- 4.20(0.86)	0.036	0.554	0.999
HIV VL -20°C for 30 days- HIV VL -20°C for 60 days	4.21(0.83)-4.20(0.86)	0.007	0.554	0.999

VL-Viral load, RT- Room temperature, SD-Standard deviation, HIV-Human Immunodeficiency virus and RNA-Ribonucleic Acid

### Viral load at different plasma storage time and temperature

There was no significant difference between mean viral load quantified at 6hrs and after 24 hours *(p = 0*.*999)* or 48 hours *(p = 0*.*430)* room temperature plasma storage. There was significant difference between mean viral load measured at 6hrs and plasma stored in 2–8°C for 15 days (*p < 0*.*001*). This significant difference was maintained when data was analyzed using Post-hoc analysis (*p = 0*.*006*). There was also significant difference between mean viral load of plasma stored in 2–8°C for 6 days and for 15days (*p< 0*.*001*) [Table 2]. However, considering the recommended clinical significance cut-off point of 0.5log, all statistically significant differences were not clinically significant since the mean differences were 0.06 log copies/ml when plasma was stored for 6 days and 0.05 log copies/ml when stored for 15 days. There was no significant difference between mean viral load at 6hrs and after storing plasma in -20°C for 30 and 60 days (*p = 0*.*576)* [Table 2].

### Viral load level at different freeze-thaw cycle and dilution

**Fig 3:**
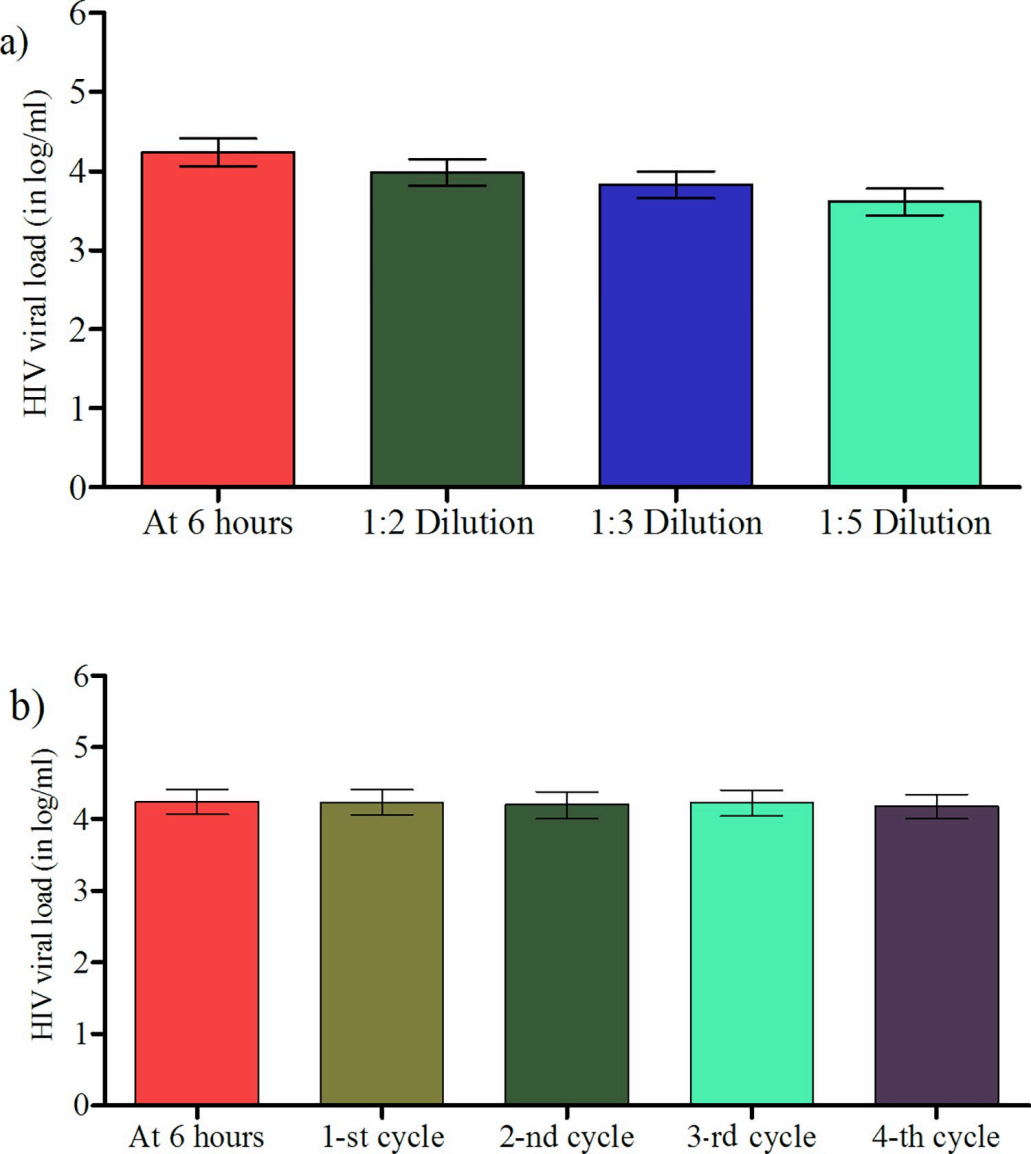
(a) HIV viral load measurement after dilution of plasma With PBS. (b) HIV viral load measurement after each of four cycles of freeze-thawing.

The mean viral load measured at 6hrs after specimen collection and from plasma freeze-thawed for four consecutive cycles after storage at -20°C, with seven days between each cycle revealed a statistically significant difference (*p = 0*.*009)* [Fig 3]. Post-hoc analysis indicated the presence of significant difference between mean viral load measured at 6hr after specimen collection and the fourth cycle of freeze-thawing (*p = 0*.*013)* [Fig 3]. However, the mean difference was 0.063 log copies/ml which was not clinically significant at 0.5 log cut-off of point. In addition, there was no significant difference between the mean of viral load for undiluted and diluted plasma (*p = 0*.*707*).

There was no statistical and clinical significant difference on dilutions proportions (a). There was a statistically significant 0.06 mean decrease after the fourth freeze-thaw cycle (p = 0.013) but it was not clinically significant (i.e. < 0.5 Log HIV RNA copies /mL) (b).

## Discussion

Several studies have focused on identifying problems related to blood collection and processing parameters that could affect accuracy and reproducibility of quantitative HIV-1 RNA viral load testing. However, there are few studies on the effects of plasma separation time, storage time and temperature, freeze-thaw cycles and dilution proportion on HIV-1 RNA viral load. Thus, this study aimed to investigate effects of sample management on HIV viral load measurement in a total of 88 participants from ART clinic in Ethiopia.

This study revealed that the mean HIV-1 RNA viral load from plasma separated at 24hrs post collection, plasma stored in 2–8°C for 15 days and at the fourth cycle of freeze-thawing were significantly different when compared to the mean viral load tested at 6hrs after collection. Although the differences were statistically significant, the changes were not clinically significant at the recommended cut-off point 0.5log copies/ml (±2SD). There was no significant mean difference in viral load at all room temperature and -20°C storage scenarios of plasma samples as compared to the viral load tested within 6hrs of collection.

Evidence indicated that HIV viral load decreases up to 0.5 log copies/ml when whole blood was stored at room temperature for 72hrs [12]. Another finding has shown that mean HIV viral load decreases significantly when a whole blood sample is stored at room temperature for 30hrs as compared to viral load tested at 2hrs [11]. This research finding was similar to our finding in which the mean HIV viral load was significantly different between viral load tested within 6hrs and after 24hrs storage of whole blood.

In contrast, other studies have found that the mean viral load was not significantly different between HIV viral load tested within 4hrs and after 24hrs storage as whole blood [7]. Furthermore, HIV-1 RNA was found to be stable up to 48hrs in whole blood at room temperature [6, 9]. Additionally, longer stability of HIV-1 RNA for up to 3 days [13] and 7 days [4] at room temperature has been reported. This difference might be due to HIV strains variation, the level of damaged HIV-1 RNA in the plasma and variation in testing conditions.

The statistically significant difference observed in our study was not clinically significant based on the cut-off point proposed by previous studies such as >±2SD or a variation of < 0.5log difference [12, 13, 21]. A previous study has reported a clinically significant difference in the mean HIV viral load when viral load was tested at 6hrs and 24hrs [12]. This finding contradicted the current study finding in which the mean viral load was not clinically significantly different between viral load tested at 6hrs and 24hrs after specimen collection. This difference could most probably be due to statistical test used, duration from sample collection to test and sample size difference. For example, the previous study used paired t-test to test the statistical difference. This test could increase type one error when used for more than two categories [30]. However, in the current study repeated measure analysis of variance was used which is free from the problem indicated above [30]. Furthermore, the study reported by Sebire *et al* [12] based on an experiment conducted on 20 participants is too small sample size compared to ours (88 participants).

The difference in the mean HIV viral load quantified from plasma stored at 2–8°C for 15 days was significantly different compared to the viral load tested within 6hrs after sample collection. This finding was in line with a previous study in which viral load was significantly different compared to the viral load tested within 2hrs and 72hrs for the plasma stored in 4°C [12]. In contrast, viral load remained stable at 4°C for up to 24 to 48hrs [6], 30hrs [5], 14 and 28 days [18]. This difference could be due to temperature fluctuation and poor temperature regulation in the room where the test was done. Although the difference in mean HIV viral load of plasma stored at 2–8°C for 15 days was significantly different in the current study, it was not clinically significant at the cut-off point of 0.5log.

The mean HIV viral load was significantly different in plasma tested after the fourth cycle of freeze-thawing after storing in -20°C as compared with the mean HIV viral load tested within 6hrs. However, the difference was not significant up to 3^rd^ cycles of freeze-thawing in comparison with mean HIV viral load tested within 6hrs. In contrast to this finding, the viral load from plasma stored in -70°C freezer and freeze-thawed for the fourth cycle was not significantly different [6]. This difference is most probably because of temperature difference and might suggest that lower storage temperature gives better result when freeze thawing is unavoidable. Although the mean difference was significant, it was not clinically significant as the mean difference was <0.5 log. Thus, care has to be taken when interpreting statistical significance as it is demonstrated in the current study where the observed statistically significant mean differences were more likely to be clinically irrelevant.

Even though SOP was followed strictly, personal variation in test procedure and the lower limit of detection of Abbott analyzer (2.2 log copies/ml for 0.2mL of plasma sample) could affect the results of this study. In our actual situation, transportation conditions would not be similar to the scenarios described in this study since the sample collection site (St Paul’s Hospital Millennium Medical College) and EPHI are within 5 minutes walking distance. Thus, the result of this study could not be equally implemented to the rural area where transportation is not easily accessed. Electric power fluctuation could also impact the freeze-thaw test result of this study. However, since the laboratory has a backup generator power, temperature fluctuation had minim effect. In the current study, -80°C freezer was not used for freeze-thawing process, which could limit our conclusion of freeze-thawing cycle at this storage temperature. Furthermore, all specimens obtained for this study were above the clinically relevant threshold for HIV-1 viral load failure (3 log copies/mL). Thus, similar studies targeting this limitation are recommended.

## Conclusion

This study has shown that HIV-1 RNA in plasma maintained stability and was not degraded by greater than 0.5log for all scenarios of the experiment. However, there was statistically significant difference on the mean viral load between viral load tested at 24hrs, stored at 2–8°C for 15 days and freeze-thawed for four cycles compared to viral load tested at 6hrs after sample collection. All the statistically significant variables were not clinically significant. Thus, to be on the safe side, plasma should be separated before 24hrs from the whole blood left at room temperature. In addition, plasma should not stay in 2–8°C beyond 15 days and should not be freeze-thawed for more than three cycles.

## Supporting information

S1 Data (SAV)

## References

[1] WHO HIV treatment and care, what’s new in treatment monitoring: viral load and CD4 testing, update July 2017 (www.who.int/hiv). Accessed on August 27/2019.

[2] WHO, Technical and operational considerations for implementing HIV viral load testing, World Health Organization 2014 (www.who.int/about/licensing//en/index.html). Accessed on February 03/2018.

[3] Doherty M, WHO guidelines on the use of CD4, Viral Load and EID tests for initiation and monitoring of ART, Treatment and Care Unit WHO,2013 Geneva (www.who.int/about/licensing//en/index.html). Accessed on February 03/2018.

[4] Vandamme A, Van Laethem K, Schmit J, Van Wijngaerden E, Reynders M, Debyser Z, et al. Long-term stability of human immunodeficiency virus viral load and infectivity in whole blood. *Eur J Clin Invest* 1999; 29(5): 445–452. doi: 10.1046/j.1365-2362.1999.00462.x 10354202

[5] Christine C, Ginocchio W, Mark H, Kaplan M, Donald W, Joseph W, et al. Effects of Specimen Collection, Processing, and Storage Conditions on Stability of Human Immunodeficiency Virus Type 1 RNA Levels in Plasma. *J*. *Clin*. *Microbiol* 1997; 35(11): 2886–2893. doi: 10.1128/jcm.35.11.2886-2893.1997 9350753 PMC230081

[6] Lew J, Reichelderfer P, Fowler M, Bremer J, Carrol R, Cassol S, et al. Determinations of Levels of Human Immunodeficiency Virus Type 1 RNA in Plasma: Reassessment of Parameters Affecting Assay Outcome. *J*. *Clin*. *Microbiol* 1998; 36(6): 1471–1479. doi: 10.1128/JCM.36.6.1471-1479.1998 9620364 PMC104860

[7] Holguin A, Willian L, Castilla J, Sariano V. Influence of time and storage conditions on plasma HIV viral load measurements. Antiviral therapy 1997; 2(4): 265–268. 11327446

[8] Anne-Marte B, Tom J, Mette S, Kirsti J, Andreas L, Arild M, et al. Overestimation of Human Immunodeficiency Virus Type 1 load caused by the presence of cells in plasma from plasma preparation tubes. *J*. *Clin*. *Microbiol* 2009; 47(7):2170–2174. doi: 10.1128/JCM.00519-09 19420166 PMC2708492

[9] Dickover R, Herman S, Saddiq K, Wafer D, Dillon M, and Bryson Y. Optimization of specimen-handling procedures for accurate quantitation of levels of Human Immunodeficiency Virus RNA in plasma by Reverse Transcriptase PCR. *J*. *Clin*. *Microbiol* 1998; 36(4):1070–1073. doi: 10.1128/JCM.36.4.1070-1073.1998 9542939 PMC104691

[10] Holodniy M, Rainen L, Herman S, Yen-Lieberman B. Stability of plasma Human Immunodeficiency Virus load in vacutainer PPT plasma preparation tubes during overnight shipment. *J*. *clin*. *Microbiol* 2000; 38(1):323–326. doi: 10.1128/JCM.38.1.323-326.2000 10618109 PMC88717

[11] Holodniy M, Mole L, Yen-Lieberman B, Margolis D, Starkey C, Carroll R, et al. Comparative stabilities of quantitative Human Immunodeficiency Virus RNA in plasma from samples collected in vacutainer CPT, vacutainer PPT, and standard vacutainer tubes. *J*. *clin*. *Microbiol* 1995; 33(6):1562–1566. doi: 10.1128/jcm.33.6.1562-1566.1995 7650187 PMC228216

[12] Kimberley S, Kate M, Sally L, Tracey M, Chris B. Stability of Human Immunodeficiency Virus RNA in blood specimens as measured by a commercial PCR-based assay. *J*. *Clin*. *Microbiol* 1998; 36(2):493–498. doi: 10.1128/JCM.36.2.493-498.1998 9466765 PMC104566

[13] Bonner K, Siemieniuk RA, Boozary A, Roberts T, Fajardo E, Cohn J. Expanding access to HIV viral load testing: A Systematic Review of RNA stability in EDTA tubes and PPT beyond current time and temperature thresholds. *PLoS ONE* 2014, 9(12): e113813. doi: 10.1371/journal.pone.0113813 25437009 PMC4249975

[14] Bruisten S, Oudshoorn P, Van Swieten P, Boeser-Nunnink B, Van Aarle P, Tondreau S, et al. Stability of HIV-1 RNA in blood during specimen handling and storage prior to amplification by NASBA-QT. *Journal of Virological Methods* 1997; 67:199–207. doi: 10.1016/s0166-0934(97)00097-9 9300385

[15] Amellal B, Murphy R, Maiga A, Brucker G, Katlama C, Calvez V, et al. Stability of HIV RNA in plasma specimens stored at different temperatures, *HIV Medicine* 2008; 9:790–793. doi: 10.1111/j.1468-1293.2008.00632.x 18754803

[16] Qin J, Das K, Kwon E, Minhas V, Swindells S, Wood C, et al. Viral load stability of an RNA virus in stabilized blood samples. *J Bioanal Biomed* 2014; 6(6):57–60.

[17] Hardie D, Korsman S, Ameer S, Vojnov L, Hsiao N-Y. Reliability of plasma HIV viral load testing beyond 24 hours: Insights gained from a study in a routine diagnostic laboratory. *PLoS ONE* 2019; 14(7):e0219381. doi: 10.1371/journal.pone.0219381 31269089 PMC6609026

[18] Jose M, Gajardo R, Jorquera JI. Stability of HCV, HIV-1 and HBV nucleic acids in plasma samples under long-term storage. *Biologicals* 2005; 33(1): 9–16. doi: 10.1016/j.biologicals.2004.10.003 15713552

[19] Fernandes H, Morosyuk S, Abravaya K, Ramanathan M, Rainen L. Evaluation of effect of specimen-handling parameters for plasma preparation tubes on viral load measurements obtained by using the Abbott Real Time HIV-1 load assay. *J*. *Clin*. *Microbiol* 2010; 48(7):2464–2468. doi: 10.1128/JCM.00083-10 20484602 PMC2897535

[20] Griffith BP, Rigsby MO, Garner RB, Gordon MM, Chacko TM. Comparison of the amplicor HIV-1 monitor test and the nucleic acid sequence-based amplification assay for quantitation of human immunodeficiency virus RNA in serum and plasma subjected to freeze-thaw cycles. *J Clin*. *Microbiol* 1997; 35(12): 3288–91. doi: 10.1128/jcm.35.12.3288-3291.1997 9399536 PMC230164

[21] Mine M, Nkoane T, Sebetso G, Sakyi B, Makhaola K, Gaolathe T. Validation of dilution of plasma samples with phosphate buffered saline to eliminate the problem of small volumes associated with children infected with HIV-1 for viral load testing using Cobas AmpliPrep/COBAS TaqMan HIV-1 test, version 2.0 (CAP CTM HIV v2.0). *Journal of Virological Methods* 2013; 194(1–2): 217–221. doi: 10.1016/j.jviromet.2013.08.031 24025342

[22] Mossoro-Kpinde C, Mboumba Bouassa R, Jenabian M, Wolyec S, Robin L, Matta M, et al. Analytical performances of Human Immunodeficiency Virus type 1 RNA-based Amplix„ Real-Time PCR platform for HIV-1 RNA quantification. *AIDS Res*. *Treat* 2016; 2016:1–12 doi: 10.1155/2016/7954810 28050283 PMC5165142

[23] Nichols Kameko, Viral Load Specimen Referral Network Report Zambia. The Nichols Group, LLC, 2016.

[24] Central Statistical Agency (CSA) [Ethiopia] and ICF. 2018. Ethiopia Demographic and Health Survey 2016: HIV Report. Addis Ababa, Ethiopia, and Rockville, Maryland, USA: CSA and ICF http://www.dhsprogram.com/pubs/pdf/FR328/FR328.pdf, or http://www.csa.gov.et/surveyreport/category/355-dhs-2016.html, accessed date February 02, 2019.

[25] Federal Democratic Republic of Ethiopia Ministry of Health, HIV Estimates and Projections for Ethiopia; 2018. Accessed date February 02, 2019.

[26] National guidelines for comprehensive HIV prevention, care and treatment Federal Ministry of Health Feb, 2017, Ethiopia. Accessed date October 15, 2019.

[27] UNAIDS. UNAIDS 90–90–90: an ambitious treatment target to help end the AIDS epidemic Geneva. Switzerland: World health organization; 2014. Accessed date October 15, 2019.

[28] Consolidated guidelines on the use of antiretroviral drugs for treating and preventing HIV infection: Recommendations for a public health approach. Geneva: World Health Organization 2013 (http://www.who.int/hiv/pub/guidelines/arv2013/en/). Accessed date October 15, 2019. 24716260

[29] Abbot (2000) Abbott m2000sp and Abbott m2000rt operational manual, Abbott molecular, List No 9k25-01.

[30] Pallant JF, SPSS survival manual: a step by step guide to data analysis using SPSS. 2-nd edition. National Library of Sydney, Australia, 2005 (www.allenandunwin.com/spss.htm). Accessed date October 15, 2019.

